# 2-deoxy-D-Glucose-(2-DG) Prevents Pathogen Driven Acute Inflammation and Associated toxicity

**DOI:** 10.1093/geroni/igaa057.3267

**Published:** 2020-12-16

**Authors:** Sanjay Pandey, Vandana Anang, Saurabh Singh, Anant Narayan Bhatt, K Natarajan, B S Dwarakanath

**Affiliations:** 1 Dr. B.R. Ambedkar Center for Biomedical Research, University of Delhi, Delhi, Delhi, India; 2 Albert Einstein College of Medicine, Bronx, New York, United States; 3 Institute of Nuclear Medicine and Allied Sciences, Delhi, Delhi, India; 4 Shanghai Proton and Heavy Ion Center of Fudan University Shanghai Cancer Center, Delhi, Delhi, India

## Abstract

Pathogen-associated molecular patterns (PAMPs) associated with viral and bacterial infections trigger multiple inflammatory pathways which may result in oxidative stress driven toxicity, tissue fibrosis organ dysfunction and ageing. Inflammatory events need high energy demands and predominantly depends on the glycolysis. Thus, energy metabolism of the inflammatory events can be targeted to reducing the magnitude of the PAMPs driven inflammation and preventing tissue toxicity. Here we propose that 2-DG, a glycolytic inhibitor, and a potential Energy Restriction Mimetic agent (ERMA) can modulate inflammatory events and can prevent the development of acute as well as chronic pathology. For this study we induced LPS (bacterial PAMP) induced endotoxemia in mice which models infection associated inflammatory acute inflammatory events, tissue damage and organ dysfunction. 2-DG fed mice (0.4% w/v in drinking water) showed reduced LPS driven oxidative stress and capillary damage in lungs. Administration of 2-DG also reduced LPS induced spike in inflammatory cytokines (TNF, IL6 and IL1β) in the BALF and serum. Lungs of 2-DG fed mice showed lesser infiltration of inflammatory cells and reduced inflammatory signaling activation. 2-DG also downregulated the ex-vivo and in-vivo migration of the PMNCs. Furthermore, 2-DG also reduced the activation of the macrophage cells (RAW264.7) which was seen with reduction and the glycolysis and increased mitochondrial functions. Our data suggest that 2-DG administration as ERMA in drinking water can prevent pathogenic exposure driven inflammatory events which may prevent acute as well as chronic inflammatory disorders.

